# Multimodal approach to portal hypertension and gastric varices before hepatic resection for hepatocellular carcinoma: a case report

**DOI:** 10.1186/s40792-020-00952-4

**Published:** 2020-07-31

**Authors:** Norifumi Harimoto, Kenichiro Araki, Ryo Muranushi, Kouki Hoshino, Kei Hagiwara, Norihiro Ishii, Mariko Tsukagoshi, Takamichi Igarashi, Akira Watanabe, Norio Kubo, Kei Shibuya, Masaya Miyazaki, Hirofumi Kawanaka, Ken Shirabe

**Affiliations:** 1 Department of Hepatobiliary and Pancreatic Surgery, Graduate School of Medicine, Gunma University, 3-39-22, Showamachi, Maebashi, 371-8511 Japan; 2 Department of Innovative Cancer Immunotherapy, Graduate School of Medicine, Gunma University, Maebashi, Japan; 3 Department of Radiation Oncology, Graduate School of Medicine, Gunma University, Maebashi, Japan; 4 Department of Applied Medical Imaging, Graduate School of Medicine, Gunma University, Maebashi, Japan; 5 Clinical Research Institute and Department of Surgery, National Hospital Organization, Beppu Medical Center, Beppu, Japan

**Keywords:** Hepatocellular carcinoma, Portal hypertension, Portal vein embolization, Balloon-occluded retrograde transvenous obliteration, Hepatic resection

## Abstract

**Background:**

Liver cirrhosis occurs in approximately 80–90% of patients with hepatocellular carcinoma (HCC), and hepatic resection may be dangerous because of well-documented liver cirrhosis, which may be accompanied by portal hypertension (PH). Here we report a patient with advanced HCC with gastric varices and PH who experienced a good clinical course after undergoing balloon-occluded retrograde transvenous obliteration (BRTO), percutaneous transhepatic portal vein embolization (PTPE), hand-assisted laparoscopic (HALS) splenectomy, and right lobectomy of the liver.

**Case presentation:**

A 72-year-old man had two HCCs with gastric varices. CT revealed one tumor (4.5 cm) located in segment 7, involving the right hepatic vein, adjacent to the middle hepatic vein. Another tumor (2.7 cm) was located in segment 6. He first underwent BRTO for gastric varices and PTPE for planned right lobectomy of the liver. To reduce PH, HALS splenectomy was performed, and uncomplicated right lobectomy of the liver was performed 10 weeks after the first visit. He has remained free of recurrence for at least 1 year.

**Conclusions:**

Our patient underwent uncomplicated BRTO, PTPE, HALS splenectomy, and right lobectomy of the liver for advanced HCC with PH. Controlling portal pressure is important when hepatic resection is required to treat HCC with PH.

## Background

Hepatocellular carcinoma (HCC) is the third most common malignancy worldwide [1]. Liver cirrhosis occurs in approximately 80–90% of patients with HCC, and hepatic resection tends to be dangerous because of liver cirrhosis [2, 3] if patients with liver cirrhosis have concomitant portal hypertension (PH). PH is pathophysiologically characterized by thrombocytopenia caused by splenomegaly, esophagogastric varices, and portosystemic shunt. In the Barcelona Clinic Liver Cancer (BCLC) staging classification, PH is identified as a contraindication for hepatic resection because of high postoperative morbidity and poor survival [2, 4, 5]. Patients with HCC with PH may be candidates for liver resection because of the safety of the procedure as well as prophylactic perioperative management that may help overcome complications after hepatic resection associated with PH [6].

Here we report a patient with a good clinical course who underwent balloon-occluded retrograde transvenous obliteration (BRTO), percutaneous transhepatic portal vein embolization (PTPE), hand-assisted laparoscopic (HALS) splenectomy, and right lobectomy of the liver for advanced HCC with PH.

### Case presentation

**Fig. 1:**
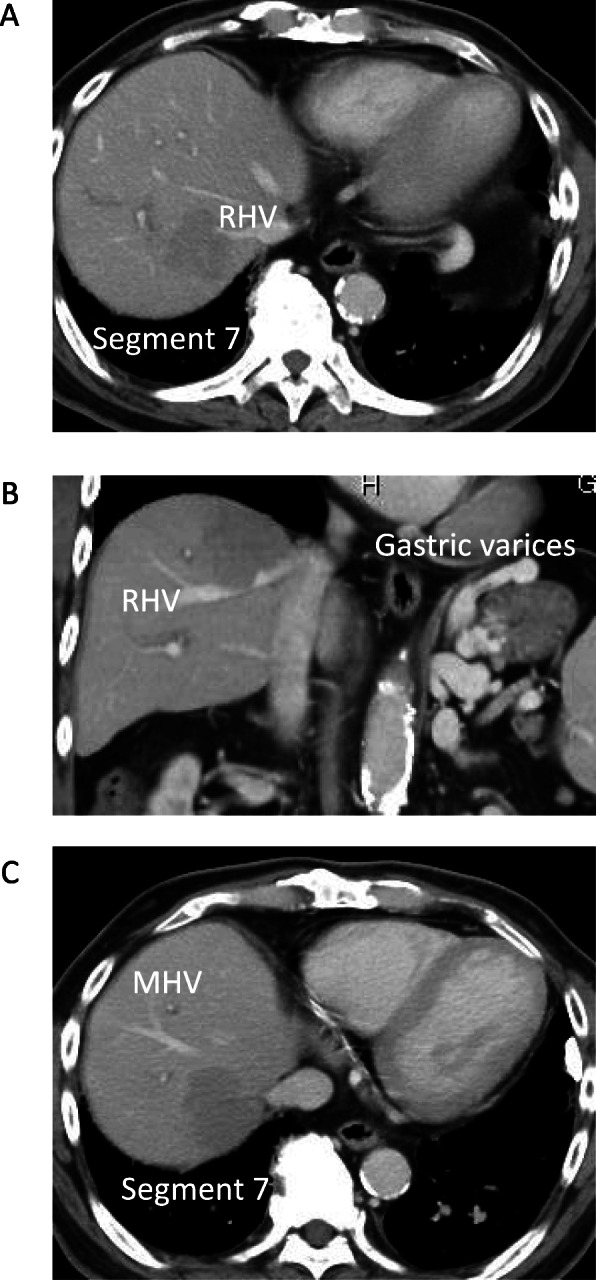
CT imaging of HCC. CT showing the tumor located in segment 7 involving the right hepatic vein, **a** transverse plane, **b** coronal plane, and **c** close to the middle hepatic vein. HCC, hepatocellular carcinoma; CT, computed tomography

**Fig. 2:**
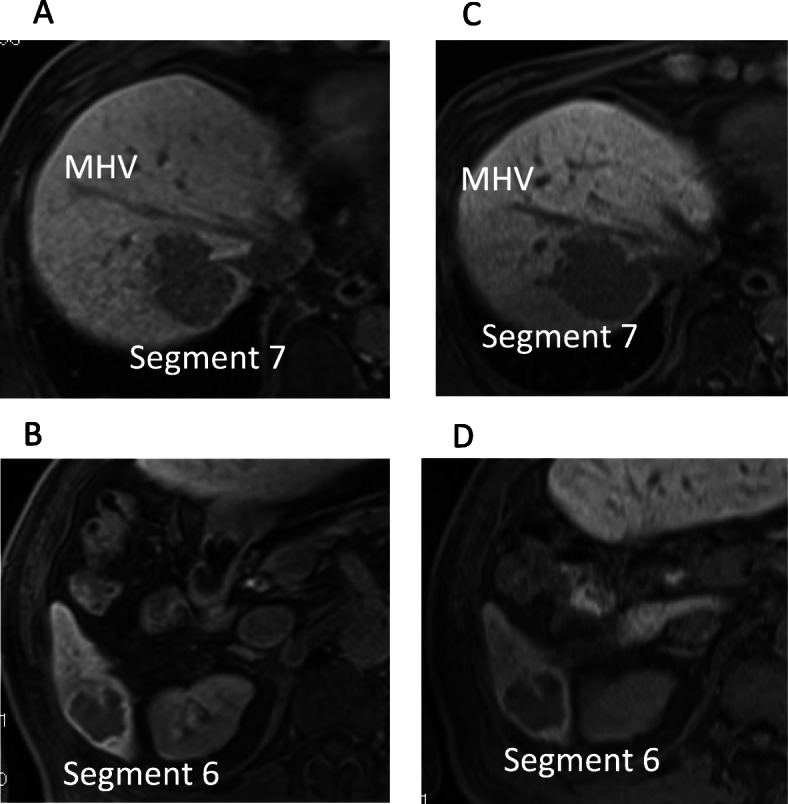
MRI imaging of HCC. **a** MRI showing the tumor located in segment 7 at the first visit. **b** MRI shows the tumor located in segment 6 at the first visit. **c** MRI shows the tumor located in segment 7 before hepatic resection. The tumor grew to 5.1 cm 10 weeks after the first visit. **d** MRI showing the tumor located in segment 6 before hepatic resection. The tumor grew to 3.1 cm 10 weeks after the first visit. MRI, magnetic resonance image; HCC, hepatocellular carcinoma; CT, computed tomography

**Fig. 3:**
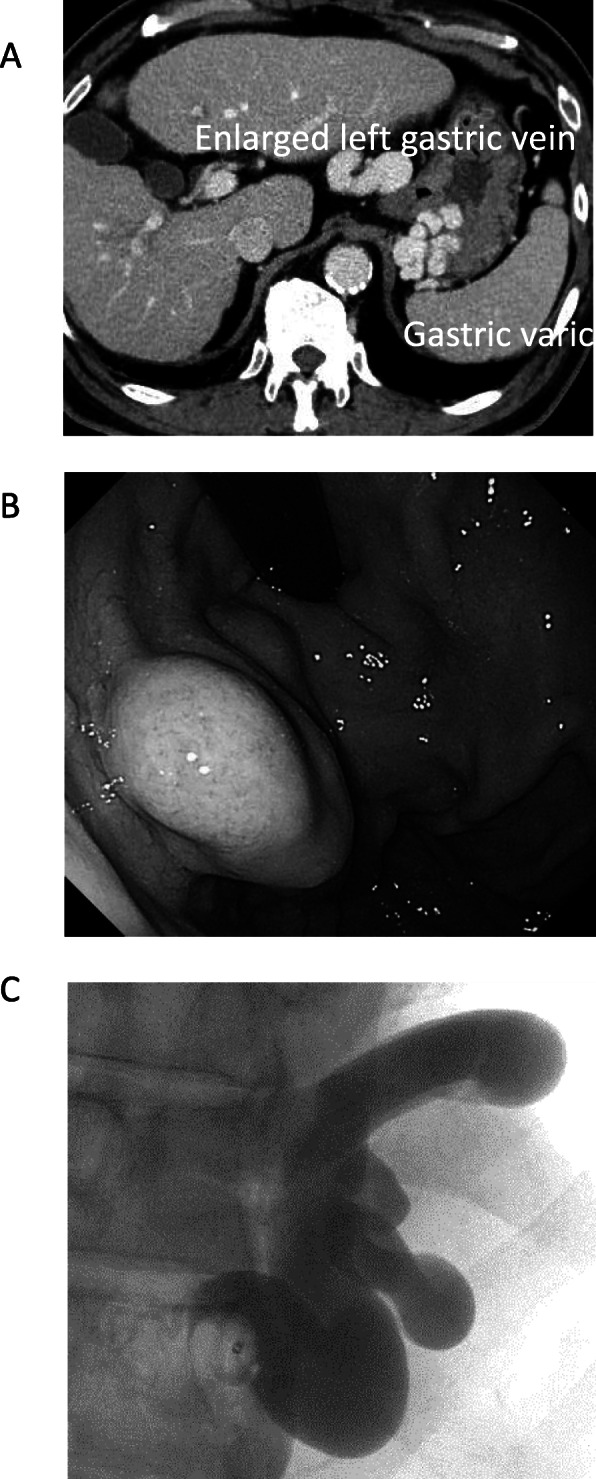
Imaging of gastric varices. **a** Gastric varices were detected by CT as an enlarged gastrorenal shunt from the left renal vein to the left gastric vein. **b** Gastric varices developed into enlarged nodules shown by upper esophagogastroduodenoscopy. CT, computed tomography

A 72-year-old man, who was diagnosed with chronic hepatitis C virus (HCV) infection, was prescribed direct-acting antivirals. After 6 months of a sustained virological response (SVR), ultrasound (US) imaging detected two liver tumors. Computed tomography (CT) revealed one tumor (4.5 cm) located at segment 7 (Fig. 1a), which involved the right hepatic vein (Fig. 1b) and was adjacent to the middle hepatic vein. A second tumor (2.7 cm) was located at segment 6. Contrast-enhanced (CE) CT detected the tumors during the early phase as well as a perfusion defect during the portal phase. Magnetic resonance imaging (MRI) revealed the same features (Fig. 2a, b). CE-CT detected gastric varices as an enlarged gastrorenal shunt from the left renal vein to the left gastric vein (Fig. 3a). Furthermore, endoscopic images showed markedly enlarged nodular (F3), risky gastric varices (Fig. 3b) [7]. ^18^F-fluorodeoxyglucose (FDG) positron emission tomography (PET) revealed the accumulation of ^18^F-FDG. The maximum standard uptake values were 5.43 at S7 and 4.42 at S6, and distant metastasis was not detected.

Laboratory results were as follows: white blood cell count, 3200/μL; platelet count, 75,000/μL; total serum albumin, 3.5 g/dL; %prothrombin time, 95%; total serum bilirubin, 0.6 mg/dL; direct bilirubin, 0.03 mg/dL; aspartate aminotransferase, 22 U/L; alanine aminotransferase, 15 U/L; alkaline phosphatase, 191 U/L; gamma-glutamyltranspeptidase, 49 U/L; ammonia concentration, 51 μg/dL; and the indocyanine green dye retention test at 15 min (ICGR15) was 15.3%. The concentrations of tumor markers such as carcinoembryonic antigen (CEA) and carbohydrate antigen (CA) 19-9 were normal, and the concentrations of arufa-fetoprotein (AFP) (217.2 ng/mL) and des-gamma-carboxy prothrombin (DCP) (1951 AU/mL) were elevated. Hepatitis B virus (HB)s-antigen, HBc-antibody, and HCV-RNA were undetectable, and HCV-antibody was positive. Mac-2 binding protein glycosylation isomer (M2BPGi) which indicated liver fibrosis was 1.95 cut off index (COI). Virtual touch quantification (VTQ) using ultrasonography was 3.09 mL/s. The Child–Pugh score was 6, grade A. The Albumin–Indocyanine Green Evaluation (ALICE) score was − 1.731 and ALICE grade was 2b.

The patient was administered insulin injection therapy because of diabetes mellitus, and he underwent percutaneous coronary intervention for coronary artery stenosis in 2015. He was diagnosed with stage III HCC with risky gastric varices. We first performed BRTO for gastric varices (Fig. 3c). The wedged hepatic venous pressure (WHVP) was 13 mmHg, and the hepatic venous pressure gradient (HVPG) was 11 mmHg. WHVP increased to 15 mmHg after BRTO. The tumor was close to the middle hepatic vein, and we therefore planned right-hepatic resection of the liver.

**Fig. 4:**
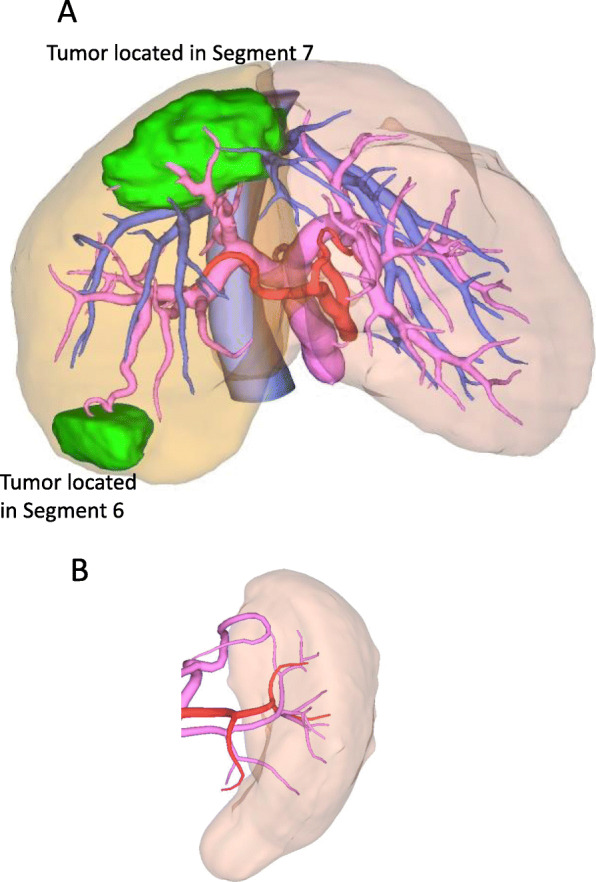
Three-dimensional CT volumetry. **a** After right-hepatic resection, the remnant liver volume was 867 mL (59.4%). **b** Spleen volume at the first visit was 428 mL. CT, computed tomography

The remnant liver volume was 867 mL (59.4%), according to 3D-CT volumetry (Fig. 4a). Although the remnant liver volume was sufficient, to reduce surgical risk, we planned PTPE and HALS splenectomy before hepatic resection because of PH. We performed PTPE 9 days after BRTO without complications. After PTPE, the portal vein pressure was 15 mmHg. Spleen volume was 428 g at the first visit (Fig. 4b) and increased to 512 g just before splenectomy. Three weeks after PTPE, HALS splenectomy was performed using an 8-cm midline incision and four ports. The spleen weighed 439 g. Operation time was 191 min, blood loss was 42 g, and blood transfusion was not required. In this patients was received anti-thrombin III (ATIII) concentrates for 3 days after splenectomy because of low ATIII activity as a prophylactic treatment. There was no portal vein thrombosis, and the patient was discharged 10 days after splenectomy without complications.

Right hepatic resection was performed 27 days after HALS splenectomy. ICGR15 improved just before hepatectomy (Table 1). Three-dimensional-CT revealed an increase in the remnant liver volume (1097 mL, 64.2%). Immediately before hepatic resection, WHVP was 13 mmHg and HVPG was 9 mmHg. M2BPGi increased to 2.36 COI. The sizes of the tumors slightly increased (5.1 cm at S7 and 3.1 cm at S6) (Fig. 2c, d), and the levels of tumor markers increased (AFP, 611 ng/mL and DCP, 3289 AU/mL). Operation time was 473 min, blood loss was 136 g, and a blood transfusion was not required.

**Table 1:** The summary of clinical course

Progress from the first visit 0	14 days		23 days		44 days		71 days	
	BRTO		PTPE		HALS splenectomy		Right lobectomy	
	Before	After	Before	After	Before	After	Before	After
Albumin (g/dL)	3.5	3.4	3.4	3.4	3.4	3.2	3.4	3.5
Total bilirubin (mg/dL)	0.6	0.6	0.5	0.6	0.5	0.6	0.6	0.7
%PT	95	102	98	102	104	108	105	92
ICGR15(%)	15.1	5.2	–	–	–	–	12.1	–
ALICE score	− 1.73	− 1.96	–	–	–	–	− 1.72	–
ALICE grade	2b	2a	–	–	–	–	2b	–
Platelet count (× 10^4^/μL)	7.6	5.7	6.5	7.9	7.2	16.3	13.8	13.9
NH_3_ (μg/dL)	51	30	41	48	39	35	35	39
AFP (ng/mL)	217.2	–	–	–	–	–	611	5.8
PIVKAII (mAU/mL)	1951	–	–	–	–	–	3289	24
Remnant liver volume (mL)	867	–	–	–	–	–	1097	–
Remnant liver volume (%)	59.4	–	–	–	–	–	64.2	–
WHVP (mmHg)	13	15	–	–	–	–	13	–
HVPG (mmHg)	11	13	–	–	–	–	9	–
PVP (mmHg)	–	–	13	15	–	–	–	–
M2BPGi (COI)	1.95	–	–	–	–	–	2.36	–
VTQ (m/s)	3.09	–	–	–	–	–	–	–

*BRTO* balloon-occluded retrograde transvenous obliteration, *PTPE* percutaneous trans-hepatic portal vein embolization, *HALS* hand-assisted laparoscopic, *PT* prothrombin time, *ALICE* Albumin–Indocyanine Green Evaluation, *ICGR15* indocyanine green dye retention test at 15 min, *AFP* α-fetprotein, *DCP* des-γ-carboxy prothrombin, *WHVP* wedged hepatic venous pressure, *HVPG* hepatic venous pressure gradient, *PVP* portal vein pressure, *M2BPGi* Mac-2 binding protein glycosylation isomer, *COI* cut-off index, *VTQ* virtual touch quantification

**Fig. 5:**
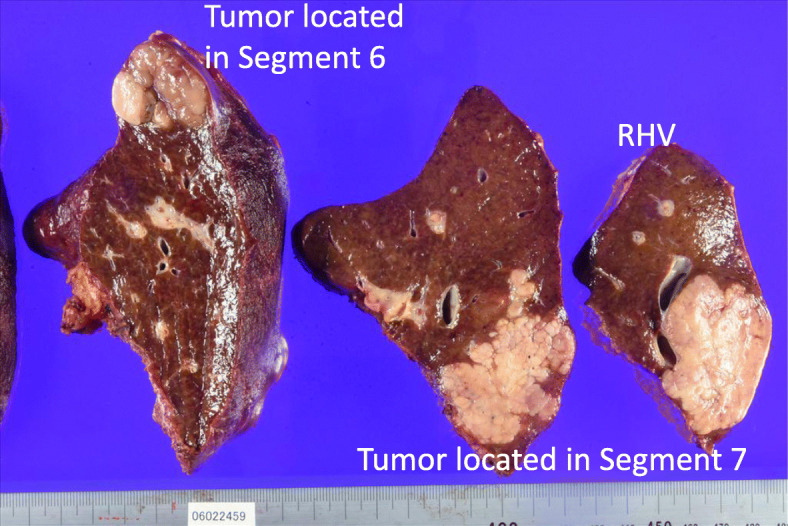
Macroscopic findings of HCC. Both tumors were confluent, multinodular-type HCC. The right hepatic vein was surrounded by the tumor, but a venous thrombus was not detected. HCC, hepatocellular carcinoma

Figure 5 shows the macroscopic findings of the resected tumors. The right hepatic vein was surrounded by the tumor, although a venous thrombus was not detected. The patient was discharged 13 days after hepatic resection without complications. Histological diagnosis was well- to moderately differentiated HCC, and portal vein invasion by the tumor was detected in S7. Fibrosis grade was F3. Table 1 summarizes the patient’s clinical course. When this manuscript was submitted, he was free of recurrence for 1 year. He received vaccination after hepatectomy to prevent over whelming post splenectomy infection (OPSI).

## Discussion

To control PVP, it is important to consider the strategy for HCC with PH. Berzigotti et al. [8] determined portal hypertension as an independent factor for decreased long­term survival and increased perioperative decompensation after resection of HCC. Bruix and Llovet evaluated the role of direct preoperative measurement of portal venous pressure to predict the outcome after liver resection in patients with cirrhosis [2, 5]. These studies found that HVPG ≥ 10 mmHg is a predictive factor for postoperative liver decompensation. Furthermore, HVPG is an indirect measurement of the actual PVP, which allows obviating risks linked with the direct puncture of the portal vein.

Evidence indicates that PVP reflects poor prognosis. For example, Chen et al. found that PVP > 16 cm H_2_O (= 11.8 mmHg) with sensitivities of 82% and specificity of 70% for predicting postoperative liver failure [9]. Hidaka et al. found that the 5-year overall survival rate and recurrence-free survival rate were significantly higher for patients with low PVP (< 20 cm H_2_O = 14.7 mmHg) compared with those with high-PVP (> 20 cm H_2_O = 14.7 mmHg) [10]. In the present case, we reduced HVPG to > 10 mmHg before hepatic resection. Sirata et al reported that in the ALICE grade 2 group, patients with PH showed high incidence of large-volume ascites and post-hepatectomy liver failure for HCC [11]. In addition, sectoriectomy or more was also a risk factor to large-volume ascites and liver failure in patients with ALICE grade 2 and PH. The ALICE score was calculated by means of the following formula: 0.663 × log10 ICGR15 − 0.718 × albumin (g/dl) [12]. The ALICE grade was stratified as follows: ALICE grade 1, linear predictor value of < − 2.20; ALICE grade 2a, linear predictor value of − 2.20 to − 1.88; ALICE grade 2b, linear predictor value of − 1.88 to − 1.39; and ALICE grade 3, linear predictor value of > − 1.39 [11, 12]. The ICGR15 has been widely used in the field of hepatobiliary surgery in Japan. Some authors have investigated the usefulness of the ALICE grade to predict liver function or prognosis [11, 12]. Bogner reported that an intraoperative PVP increase was an independent predictor of post-hepatectomy liver failure after major hepatectomy [13]. In our case, PVP was increased by both BRTO and PTPE, the complication after the right lobectomy will be strongly expected. We conclude therefore that we employed an appropriate strategy for treating this patient’s HCC with PH to maximally reduce surgical complications.

Evaluating the degree of liver fibrosis before surgery is also important. Liver biopsy not only is a very high-risk procedure to diagnose liver fibrosis but also has a limited indication. Several reports have focused on the relationship between M2BPGi or VTQ and liver fibrosis. M2BPGi and VTQ were reported to predict liver fibrosis precisely compared with markers such as hyaluronic acid and type IV collagen [14, 15]. According to the cut-off value of M2BPGi and VTQ [14, 15], the fibrosis of these patients could be predicted fibrosis 3 or 4.

On the other hand, liver fibrosis and portal hypertension after HCV eradication will improve, but it takes much time [16]. The degree of improvement depends on baseline HVPG. Among patients with HVPG of 10–15 mmHg, PH resolved in 43% after HCV eradication, but PH did not resolve among patients with HVPG ≥ 16mmHg [16]. This patient who had risky gastric varices and low platelet count would have tolerated hepatectomy even without splenectomy considering SVR.

Liver transplantation is an ideal treatment for patients with HCC with PH; however, donor shortages and older recipients mean that candidates for liver transplantation are inevitably limited. Among treatment options, which is best, anatomical or nonanatomical resection? Is synchronous splenectomy better? [17–19]. In any case, modulating portal vein pressure is essential for patients who undergo hepatic resection because PVP is significantly associated with short- and long-term prognoses.

BRTO is a reliable and safe procedure for radical treatment of gastric varices, which are related to portosystemic shunt [20]. BRTO increases hepatopetal portal flow and improves liver function of patients with reserve liver capacity [21]. High splenic volume is an independent predictor of the recurrence of posthepatectomy HCC and overall survival [22]. Thus, splenectomy reduces portal pressure in patients with PH and improves liver function [23]. Although portal vein thrombosis is a postoperative complication of splenectomy, recent advances in laparoscopic surgery and the treatment of PV thrombosis increase safety and reduce the invasiveness of splenectomy when administered to patients with liver cirrhosis and PH [24]. Kawanaka et al. found that HVPG significantly increases after B-RTO (average increase, 27.6%), although HVPG tends to decrease (average reduction, 14.8%) after BRTO combined with splenectomy [25].

Splenectomy reduces PH, but there were some problems such as portal vein thrombosis and OPSI. Cirrhotic patients after splenectomy show decreased levels of ATIII activity, which are associated with hypercoagulable status, and reduced portal venous flow, resulting from the elimination of increased splenic blood flow. This has been found to amplify the incidence of PVT considerably, as much as 24 to 36% [26, 27]. Kawanaka et al. reported that ATIII is recommended for patients at high risk for thrombotic complications, including splenectomy in patients with liver cirrhosis, and is safe and effective prophylactic methods that do not increase the risk of bleeding [26]. OPSI is a syndrome of fulminant sepsis occurring in splenectomized patients that are associated with high mortality and morbidity [28]. The pneumococcal vaccination will be mandatory.

To reduce the surgical complication especially such as liver failure or refractory ascites, remnant liver volume is important. PTPE induced significant compensatory hypertrophy whether in the non-cirrhosis group (*p* = 0.002) or cirrhosis group (*p* < 0.001); however, no significant difference was identified between the two groups, with respect to left liver volume enlargement 4–6 weeks following PVE (*p* = 0.373) [29]. On the other hand, PTPE needs waiting time for hypertrophy, which allows tumor progression.

Certain interventions for PH are time-consuming, which allows tumor progression. In the present study, although the levels of the tumor markers AFP and DCP were elevated and tumors were slowly growing, hepatic resection was fortunately performed 10 weeks after the first visit without complications. To measure PVP, WHVP, or HVPG, as performed here, this case may be helpful for indicating hepatic resection. Thus, if PVP before hepatic resection exceeds 15 mmHg, partial hepatic resection should be selected.

## Conclusions

Our experience with the present patient who underwent multiple procedures to treat advanced HCC with PH provides compelling evidence that controlling PVP is important when the hepatic resection is required for treating this condition.

## Data Availability

Data sharing is not applicable to this article as no datasets were generated or analyzed during the current study.

## Abbreviations

CT: Computed tomography
CE-CT: Contrast-enhanced CT
US: Ultrasound sonography
MRI: Magnetic resonance imaging
^18^F-FDG-PET: 2-Deoxy-2-[fluorine-18]fluoro-D-glucose integrated with computed tomography
ICGR15: Indocyanine green dye retention test at 15 min
HVPG: Hepatic venous pressure gradient
WHVP: Wedged hepatic venous pressure
HCC: Hepatocellular carcinoma
PVE: Portal vein embolization
BRTO: Balloon-occluded retrograde transvenous obliteration
AFP: α-Fetprotein
DCP: des-γ-carboxy prothrombin
